# Artificial Intelligence and Its Theranostic Applications in Dentistry

**DOI:** 10.7759/cureus.38711

**Published:** 2023-05-08

**Authors:** Karthik Rajaram Mohan, Saramma Mathew Fenn

**Affiliations:** 1 Oral Medicine, Vinayaka Mission's Sankarachariyar Dental College, Vinayaka Mission's Research Foundation (Deemed to be University), Salem, IND; 2 Oral Medicine and Radiology, Vinayaka Mission's Sankarachariyar Dental College, Vinayaka Mission's Research Foundation (Deemed to be University), Salem, IND

**Keywords:** neural network, deep learning, machine learning, dentistry, artificial intelligence

## Abstract

As new technologies emerge, they continue to have an impact on our daily lives, and artificial intelligence (AI) covers a wide range of applications. Because of the advancements in AI, it is now possible to analyse large amounts of data, which results in more accurate data and more effective decision-making. This article explains the fundamentals of AI and examines its development and present use. AI technology has had an impact on the healthcare sector as a result of the need for accurate diagnosis and improved patient care. An overview of the existing AI applications in clinical dentistry was provided. Comprehensive care involving artificial intelligence aims to provide cutting-edge research and innovations, as well as high-quality patient care, by enabling sophisticated decision support tools. The cornerstone of AI advancement in dentistry is creative inter-professional coordination among medical professionals, scientists, and engineers. Artificial intelligence will continue to be associated with dentistry from a wide angle despite potential misconceptions and worries about patient privacy. This is because precise treatment methods and quick data sharing are both essential in dentistry. Additionally, these developments will make it possible for patients, academicians, and healthcare professionals to exchange large data on health as well as provide insights that enhance patient care.

## Introduction and background

Artificial intelligence (AI) is the use of computers and technology to simulate human-like intelligent behaviour and critical thought. John McCarthy, who has been hailed as the father of artificial intelligence, was the first to use the term artificial intelligence [1]. AI refers to a broad spectrum of technologies that continue to influence our daily lives as new ones are developed. The development of artificial intelligence has made it feasible to analyse vast amounts of data, leading to more accurate data and better decision-making. The foundations of AI are discussed in this article, along with an analysis of its history and current practical applications in dentistry. Due to the demand for precise diagnosis and enhanced patient care, artificial intelligence technology has had an effect on the healthcare industry. A summary of the current applications of artificial intelligence in clinical dentistry was given. Artificial intelligence in health care is used to record medical reviews, interpret radiological images, and make clinical diagnoses and treatment plans. By providing sophisticated decision support tools, comprehensive care, including artificial intelligence, intends to provide cutting-edge research and developments as well as high-quality patient care. Creative interprofessional collaboration between medical professionals, scientists, and engineers is the cornerstone of AI advancement in dentistry. Despite possible misunderstandings and concerns about patient privacy, artificial intelligence will continue to be involved with dentistry on a broad scale. This is due to the importance of precise treatment techniques and efficient health-related data exchange in dentistry. These advancements will also enable the transmission of vast amounts of health-related data between patients, academics, and healthcare professionals, as well as the provision of insights that improve patient care [1].

Methodology

**Figure 1:**
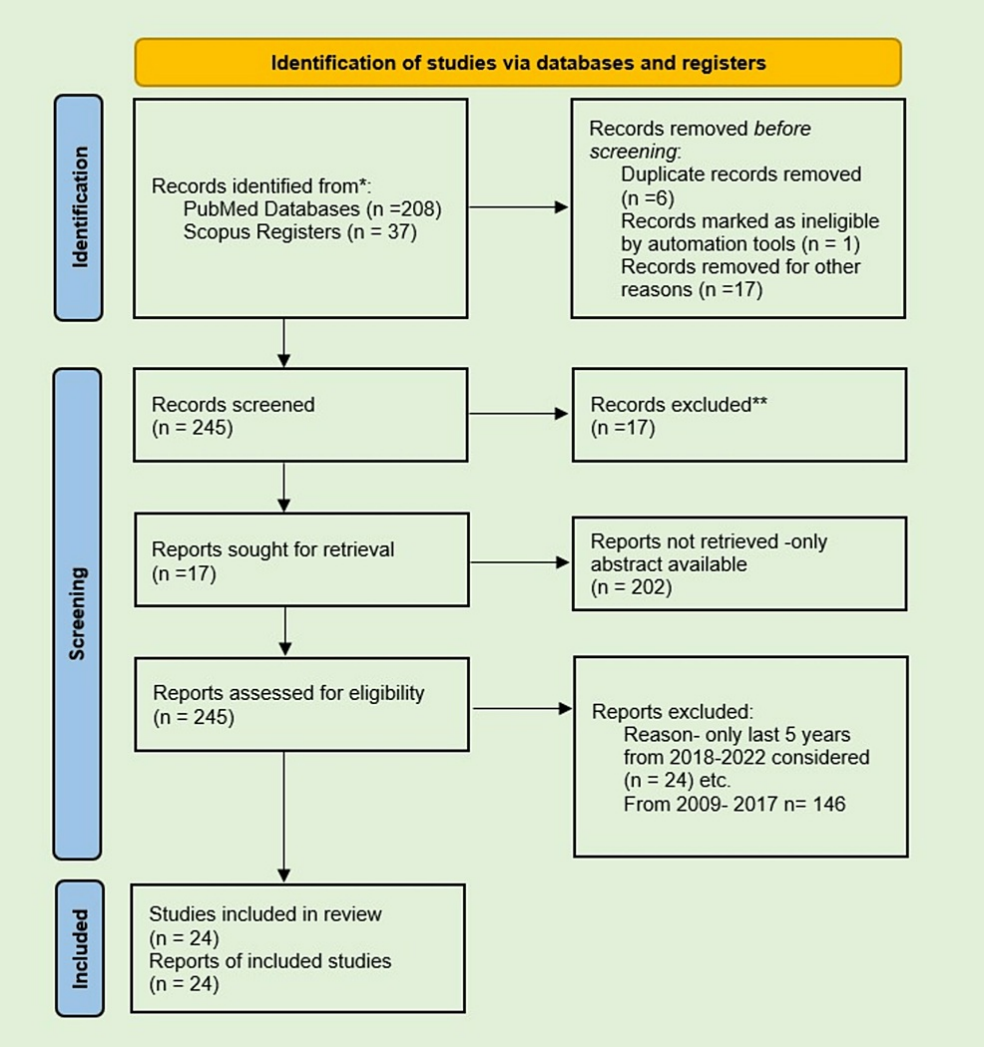
Literature search on artificial intelligence in dentistry using PRISMA strategy.

A search of PubMed and Scopus-indexed journals for the last five years (2018-2022) using the phrase 'Artificial intelligence in Dentistry' in PubMed and Scopus's databases. The findings revealed 208 publications indexed by PubMed and 35 publications indexed by Scopus on artificial intelligence in dentistry. 202 publications are not retrieved, only abstracts are available, and duplicate publications are excluded. Only 24 research studies were considered for a systematic review following PRISMA Strategy screening from 2018 to 2022 (Figure 1).

## Review

Artificial intelligence is the use of computers and technology to simulate human-like intelligent behaviour and critical thought [1].

Types of artificial intelligence

The capacities and functionalities of artificial intelligence can be categorised. There are three types of artificial intelligence, based on capabilities: general artificial intelligence, targeted artificial intelligence, and super artificial intelligence. The four types of artificial intelligence that fall under the functionalities category are limited theory, reactive machines, self-awareness, and theory of mind [1].

Narrow artificial intelligence

Narrow artificial intelligence, often referred to as weak artificial intelligence, focuses on a particular task and is unable to do tasks outside of its limitations. It advances along the spectrum of one particular category of cognitive abilities. As machine learning (ML) and deep learning techniques progress, narrow artificial intelligence applications are becoming more prevalent in our daily lives, e.g., Siri from Apple, Google Translate, spam filtering, and image recognition software [1].

General artificial intelligence

Strong artificial intelligence, often referred to as general artificial intelligence, is able to comprehend and learn any intellectual task that a human is able to. It enables a computer to use knowledge and skills in a variety of situations. It enables a computer to apply information and abilities in many circumstances, Tianhe-2, for example [1].

Tianhe-2 is a supercomputer built by the National University of Defense Technology in China. It packs a 33.86 petaflops cps (calculations per second) record (quadrillions of cps) when compared to the human brain, which is only capable of one billion cps [1].

Artificial intelligence is the process of generating intelligent devices from vast amounts of data. Systems learn from previous experiences and learn to accomplish human-like tasks. It increases the effectiveness, efficiency, and efficiency of spheres of human activity. Artificial intelligence uses advanced, sophisticated algorithms and construction techniques to create robots that can decide for themselves. Machine learning and deep learning are the cornerstones of artificial intelligence [1].

Super artificial intelligence

Super artificial intelligence is capable of executing any task better than a person and outperforming human intelligence. The goal of artificial superintelligence is for artificial intelligence to become so comparable to human emotions and experiences that it not only comprehends human feelings and experiences but also develops its own emotions, needs, beliefs, and goals. Its existence is still speculative. Super artificial intelligence's core capabilities include thinking, solving puzzles, formulating judgments, and making decisions independently [1].

Functionality-based AI

Limited Memory

To make decisions, artificial intelligence learns from past data. These systems' memories are transient. For a brief period of time, people can use this historical information, but they cannot add it to their experience library. This technique is employed in self-driving cars [1].

Reactive Machines

The simplest form of artificial intelligence is a reactive machine, which does not retain memories or draw on past experiences to forecast future behaviour. It only functions with up-to-date data. They remain aware of and react to their environment. Reactive machines are given certain tasks and are constrained to accomplishing them [1].

Theory of Mind Artificial Intelligence

Theory of mind artificial intelligence is a complex form of technology that only exists in conception. Such artificial intelligence requires a thorough understanding of how objects and people in an environment might affect emotions and behaviour. It should be able to interpret people's feelings, thoughts, and emotions. This area of artificial intelligence has seen tremendous improvements, but it has not yet reached its full potential [1].

For instance, in the late 1990s, a researcher at the Massachusetts Institute of Technology built the robot head Kismet. Kismet is able to mimic and identify human emotions. Kismet cannot follow gazes or transfer attention to people, despite the fact that these abilities represent substantial advancements in the theory of mental artificial intelligence [1].

Self-awareness artificial intelligence

Self-awareness artificial intelligence only exists in theory. These systems are capable of understanding both human emotions and their internal properties, moods, and circumstances. These machines will possess intelligence beyond that of humans. This kind of artificial intelligence will have its own emotions, needs, and beliefs in addition to being able to recognise and elicit the emotions of those with whom it interacts [1].

**Figure 2:**
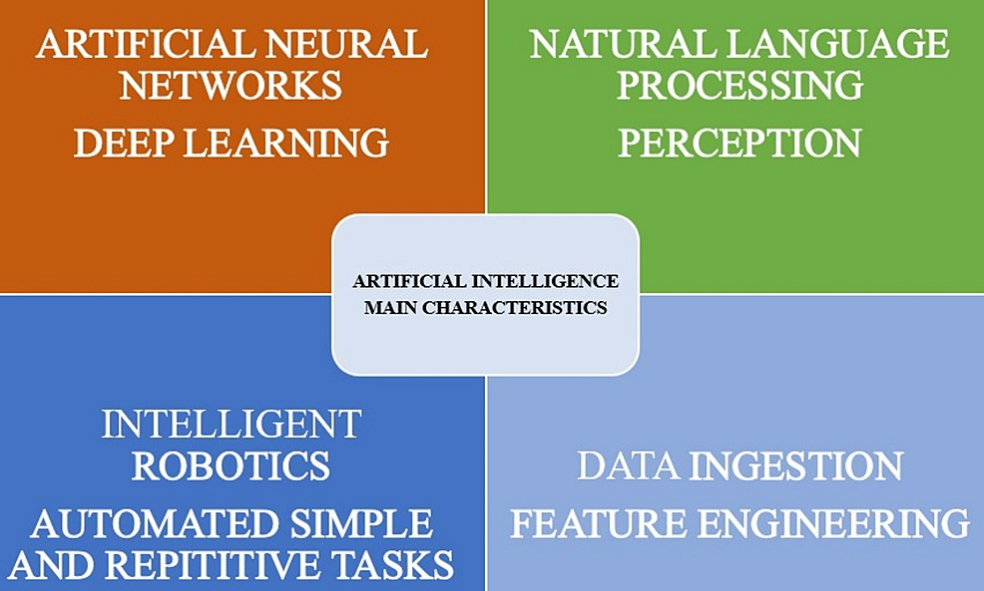
The main characteristics of artificial intelligence. Image courtesy: Thomas T. Nguyen, DMD, MSc, FRCD(C); Naomie Larrivée; Alicia Lee; Olexa Bilaniuk, BASc, MSc; Robert Durand, DMD, MSc, FRCD(C). Proper attribution and permission was obtained from the original publisher.

The main characteristics of artificial intelligence are described (Figure 2).

Artificial intelligence principle

Machine learning, a subset of AI, is the process by which computer systems learn to carry out intelligent activities without the use of pre-programmed rules or past knowledge. Instead of requiring human intervention, the systems analyse large datasets of cases to identify trends. To do this, a goal must be set, and the system's customizable functions must be optimised to help the goal be accomplished. A machine learning algorithm gains proficiency in this process, known as training, by being exposed to random cases and gradually changing the "tunables" in the direction of the correct response. The programme thus finds patterns that may be applied to brand-new pictures. This approach is comparable to an adult presenting numerous dog pictures to a young child. The toddler eventually picks up the patterns needed to recognise a dog and recognise one in fresh pictures.

In deep learning (DL), a kind of machine learning, systems aim to learn incremented gradually related, composable patterns, not just a single pattern. A "deep" system is produced by combining and stacking patterns and is substantially more effective than a straightforward, "shallow" system. A young kid might first focus on an object's edges, where a certain arrangement of them creates a textured outline with basic features like eyes and ears. This is different from how an older child recognises a dog, which involves a single, discrete pattern-matching phase. These parts begin to join together in larger groupings, such as heads and legs, and together, these larger groups make up the full dog.

**Figure 3:**
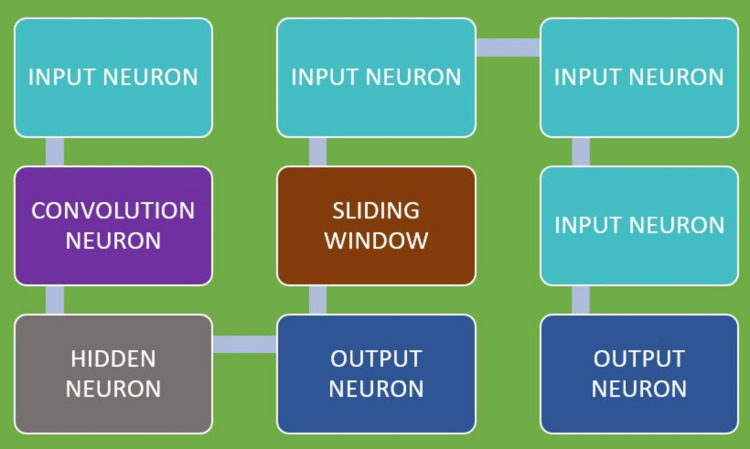
Schematic representation of deep learning in artificial intelligence by neural network. Image courtesy: Thomas T. Nguyen, DMD, MSc, FRCD(C); Naomie Larrivée; Alicia Lee; Olexa Bilaniuk, BASc, MSc; Robert Durand, DMD, MSc, FRCD(C). Proper attribution and permission was obtained from the original publisher.

A relatively well-liked family of deep learning algorithm techniques is the neural network, which is an artificial (ANN) structure comprised of several neurons, tiny units which communicate with each other and are arranged in layers. Input, output, and hidden layers are the three layers that make up a neural network. A shallow neural network (SNN) may have one or a few hidden layers, whereas a deep neural network (DNN) may have several hidden levels. Since the values of these levels are neither predefined nor obvious to outsiders, they are known as hidden layers. Artificial intelligence aims to make it possible to evaluate the accurate value of the output layer, which is visible by building gradually little by little on the information gathered from the input layer, which is also visible. The organisation of the pattern of connections between neurons determines the architecture of the neural network, and the tunable fine intensities of those connections are referred to as neural network weights (Figure 3).

In dentistry, convolutional neural networks (CNNs) are among the most frequently employed subclasses of artificial neural networks (ANN). In order to comprehend digital data, such as music, pictures, and videos, CNNs use specialised neuron connection architectures and the convolutional mathematical function. CNNs analyse a larger image or signal by scanning a relatively smaller area of neighbourhood inputs at a period of time from left extending till right and in a sequential fashion from top extending till bottom in a consequetive manner using a sliding window. They are the most popular image recognition algorithms because they are so effective at categorising images.

Advantages of artificial intelligence

Tasks are completed in nearly no time, and decisions are logical and possible, resulting in a correct diagnosis. Standardization of procedures is possible.

Disadvantages of artificial intelligence

Ample training is necessary due to the complexity of mechanisms/systems and the expensive setup. Due to the frequent use of data for training and testing in artificial intelligence, 'snooping bias' in data develops. Dental artificial intelligence findings are not directly applicable to the absence of human emotions due to the non-participation of humans.

Oral diagnosis

Detection of Dental Plaque

You et al. used an artificial intelligence neural network model to evaluate 98 intraoral pictures of baby teeth for the presence of dental plaque [2].

Diagnosis of Gingivitis

Revilla-León et al. used artificial intelligence to diagnose gingivitis from intraoral photographs with an accuracy between 74% and 78.20% and also by using fluorescent intraoral images to diagnose gingivitis with 67.7% to 73.72% accuracy [3].

Diagnosis of Odontogenic Tumours

Ameloblastomas and keratocystic odontogenic tumours, two significant maxillary tumours with distinct clinical characteristics but with similar morphological variations, were distinguished using a CNN algorithm created by Poedjiastoeti and Suebnukarn in 2018. The diagnosis accuracy and specificity of the algorithm were 83.3 and 81.8 percent, respectively, matching clinical professionals' 83.8% and 81.1%. The difference in diagnostic time, however, was more significant: convoluted neural networks were capable of diagnosis in 38 seconds compared to professionals, who needed an average of 23.1 minutes [4].

For the Diagnosis of Maxillary Sinusitis

For the objective of diagnosing maxillary sinusitis, Kim et al. examined 200 water view radiographs to determine the frequency of maxillary sinusitis using artificial intelligence and CNN [5].

For the Diagnosis of Osteoporosis

Lee et al. diagnosed osteoporosis in 200 panoramic radiographs using an artificial neural network using a DCNN-based CAD system [6].

Diagnosis of Sjogren’s Syndrome on CT Images

Sjogren's syndrome was identified by Kise et al. using artificial neural networks on computed tomography (CT) images. The deep learning system's accuracy, sensitivity, and specificity were 96.0%, 100%, and 92.0%, respectively, demonstrating its excellent power as a diagnostic tool for the identification of Sjogren's syndrome [7].

Assessment of Risk of Oral Cancer

Welikala et al. used two deep learning-based computer vision approaches to classify oral lesions, which applied image classification with ResNet-101 and object detection with the Faster R-CNN to more than 2,000 images managed by artificial intelligence with an accuracy of 87% for detecting the presence of oral lesions, 41% in the detection of oral cancer, and the 78% accuracy needed for referral treatment [8].

Dental radiology

Recognizing, Identifying, and Numbering Teeth

Convoluted neural networks (CNNs) have exhibited promise in detecting and identifying anatomical structures. In recognizing, identifying, and naming teeth from intraoral periapical radiographs, a precision rate of 95.8-99.45% was obtained by CNNs, closely matching the performance of clinical specialists, who had a precision rate of 99.98 percent [9].

To Diagnose Dental Caries

According to Lee et al., convolutional neural networks are also used to detect and diagnose dental cavities [6]. In 3000 periapical radiographs of posterior teeth, a deep CNN algorithm identified carious lesions with a sensitivity of 74.5-97.1% and an accuracy of 75.5-93.3%. With sensitivity ranging from 19% to 94%, this is a considerable advancement above clinical diagnosis using radiographs alone. When combined with their speed, deep CNNs have a greater and increased potential for improving the sensitivity of dental caries diagnosis, making them one of the most essential instruments in this field [9]. Kuhnisch et al. detected dental caries on 2,417 anonymized, high-quality intraoral clinical photographs from 1,100 permanent smooth surfaces (anterior teeth and canines = 734; posterior teeth = 366) and 1,317 permanent occlusal surfaces using artificial intelligence and were categorised by MobileNet V2 in connection with the python library [10].

To Diagnose Prediction of Root Caries

Hung et al. used machine learning for the diagnostic prediction of root caries. Support vector machine (SVM), one of the machine learning algorithms created, performed the best, diagnosing root caries with 97.1% accuracy, 95.1% precision, 99.6% sensitivity, and 94.3% specificity. The factor most significantly linked to root caries was age [11].

To Diagnose TMJ Osteoarthritis

Lee et al. developed an artificial intelligence model using the TMJOA artificial intelligence model in 3,514 sagittal CBCT pictures of the mandibular condyle from the temporomandibular joint. According to image analysis criteria for the diagnosis of temporomandibular disorder, the region of interest (condylar head) was identified and divided into two groups: uncertain for TMJOA and TMJOA. The model was tested with two sets of 300 images in total. The average precision, accuracy, recall, and F1 score over the two test sets were 0.85, 0.86, 0.84, and 0.84, respectively. Automated detection of TMJOA from sagittal CBCT images is possible by using a deep neural network model. It could help clinicians with TMJOA diagnosis and treatment decision-making [12].

Orthodontics

To Predict Mandibular Morphology in Skeletal Malocclusions

Demircan et al. predicted skeletal class III malocclusion using profile photographs by artificial intelligence [13].

Real et al. stated that ANN has enormous potential and aids in decision-making in clinical practice. To get predictable outcomes for patients, orthodontic treatments must be meticulously planned. On the other hand, tooth extractions are not uncommon in orthodontic treatment plans. As a result, it is critical to make the best clinical judgement before beginning irreversible operations. An ANN was used to assess whether tooth extraction was necessary for malocclusion patients before starting orthodontic treatment. The accuracy of the four developed ANNs, which considered numerous clinical indices, ranged from 80% to 93% when determining if extractions were necessary to treat a patient's malocclusions. A network analysis based on a Bayesian model was used to find correlations between several factors affecting the diagnosis and outcome of treatment for ectopically placed maxillary canines. In order to find correlations between these factors, all pretreatment, posttreatment, and patient-related data from the patients were gathered in this study. The study suggests that using artificial intelligence in dentistry could benefit dental professionals [14].

For Automated Identification of Cephalometric Landmarks

Kunz et al. used artificial intelligence in the identification of cephalometric landmarks from 50 cephalometric images recorded on a Sirona Orthophos XG (Dentsply Sirona, Bensheim, Germany). He concluded that artificial intelligence was on par with 12 dentists (six orthodontists and six postgraduates) in the identification of cephalometric landmarks [15].

Park et al. used artificial intelligence for the identification of cephalometric landmarks in cephalometric images [16].

To Assess Growth and Development by Assessing Cervical Vertebrae

Kok et al. examined 300 cephalometric radiographs of individuals aged 8 to 17 years. Twenty different linear measurements were made at 19 reference points on the fourth, third, and second cervical vertebrae. The selection and comparison of seven artificial intelligence categorization algorithms. k-NN, Naive Bayes, ANN, SVM, decision trees, random forests (RF), and logistic regression are some of the methods used (Log.Regr.). The highest accuracy algorithms in identifying stages of cervical vertebrae, according to the decision tree confusion matrices, were CSV1 (97.1%) - SVM: CVS4 (58.5%), 2 CVS3 (73.%), kNN: CVS6 (78.7%), CSV2 (90.5%), and CVS 5 (60.9%). The ANN algorithm was demonstrated to have the values that are accurate and is the highest but second in recognising all stages except CVS5 (93%, 68.8%, 89.7%, 55.6%, and 78%, respectively; 47.4%, the third highest accuracy value). Based on the average rank of the algorithms in predicting CSV classes, ANN had the highest level of stability, with a 2.17 average rank [17].

Oral maxillofacial surgery

For Predicting Postoperative Facial Swelling Following Mandibular Third Molar Extraction

Using artificial neural networks, Zhang et al. investigated the postoperative face swelling following surgical extraction of the mandibular third molar. He concluded that, based on the enhanced conjugate grades, the artificial neural networks BP algorithm, a newly created approach that can aid in predicting the postoperative swelling that follows the extraction of impacted mandibular third molars, has a high prediction accuracy [18].

To Assess the Impact of Facial Attractiveness After Orthognathic Surgery

Patcas et al. performed a longitudinal retrospective single-center analysis; pictures taken before and after treatment were gathered from 146 consecutive orthognathic patients (n = 2164). Data pertaining to patients were annotated on each picture (age; gender; type of malocclusion; surgery performed). Facial attractiveness was scored from 0-100 and approximate age was calculated for each image using specialised convolutional neural networks trained on more than 0.5 million photos for estimation of age and on more than 17 million assessments for attractiveness. The post and pre-treatment pictures for each patient were averaged separately, and the age, which was apparent, and real age were compared with facial appearance. An examination of changes in facial attractiveness and appearance was done statistically. The entire sample was analyzed as well as the subgroups (gender; malocclusion; type of surgery performed). The algorithms showed that the majority of patients' facial appearance improved with therapy (66.4%), notably after profile-altering surgery, and resulted in a youthful appearance of almost one year [change of mean duration: 0.93 years (95% confidence interval, CI): 1.50; 0.36; p = 0.002]. Orthognathic treatment, particularly after lower jaw surgery, showed a similar favourable effect on aesthetics in 74.7% [difference of mean: 1.22 (95% CI: 0.81; 1.63); <0.001]. This study shows how artificial intelligence can be used to assess perceived age and facial attractiveness in people with orthognathic jaw issues [19].

Periodontics

Lee et al. evaluated the effectiveness and precision of deep convoluted neural network algorithms for the prediction and diagnosis of periodontally compromised teeth. The accuracy of the CNN approach for predicting the need for tooth extraction was accurately determined at 73.4-82.8% and those that were diagnosed as periodontally compromised teeth was 76.7-81.0%. Premolars were categorised as periodontally compromised teeth more accurately than molars in terms of the observed accuracy difference between the two teeth (accuracies were 73.4% for molars and 83.8% for premolars, respectively). Premolars normally have a solitary root or roots fused, whereas molars typically have more anatomically complex morphological variations of two or three separate roots, creating a more complex anatomical structure for a CNN to comprehend [20].

Endodontics

In the 1960, 1970, and 1980 clusters, the average median survival time (MST) of composite resin, amalgam, and GIC restorations for the occlusal surface was 13.6, 16.8 years, and 7.9 years, respectively, according to Sukegawa et al. The median survival time for composite resin and glass ionomer restorations on the occlusal surface was 7.3 years and 4.9 years in the 1980 and 1970 clusters, respectively. The dental professional's primary duty was to gather and arrange the data, and the observations were collected utilising data mining methodology [21].

Prosthodontics

For Classification of Dental Implant Systems

A deep neural network was built by Sukegawa et al. for the classification of dental implant systems. Deep CNNs and transfer-learning methods were utilised to categorise and distinguish the accuracy of brands of various implant systems in dentistry using panoramic X-ray pictures [21]. Totally, 8859 implant images were gathered from 11 implant systems for objective labelling and digital panoramic radiographs of patients who received dental implant therapy at Kagawa Prefectural Central Hospital in Japan between 2005 and 2019. The VGG19 and VGG16 transfer-learning models, as well as significantly modified VGG19 and VGG16, were among the five deep CNN models that were tested for implant classification, along with a straightforward CNN with three convolutional layers. The highly calibrated VGG16 model performed better than the other four models in terms of implant categorisation. The typical transfer-learning VGG16 came in second, followed by the precisely designed VGG19. The 11 distinct types of panoramic X-ray images were successfully categorised by the properly calibrated VGG19 and VGG16 CNNs as dental implant systems [22]. Revilla-Leon et al. used artificial intelligence for tooth-supported removable and fixed prostheses [22,23].

Forensic odontology

Gender Determination

Patil et al. used artificial intelligence by analyzing morphometric parameters such as bicondylar width, condylar height, and bimental width in the mandible among 1000 digital orthopantamographic images for gender determination. Artificial intelligence using deep neural networks was found to have 75% accuracy than logistic regression models, which had 69.9% accuracy, and discriminant analysis, which had 69.1% accuracy [23].

Age Estimation by Assessing Third Molar Eruption

Age was determined by the eruption of the third molar. Automatic stage recognition was done with the help of MATLAB R2017a's many machine learning techniques [24].

Despite numerous studies suggesting possible applications for AI in dentistry, these systems are still very far from being able to replace dental specialists. AI should be seen as an extra tool that can help specialists and dentists. In order to guarantee that humans can still oversee treatment and make informed decisions in dentistry, AI must be introduced in a safe and controlled manner.

Limitations of artificial intelligence

The main limitations of artificial intelligence are the transparency of the programmed algorithm, limited accessibility, the possibility of bias and data security breaches, the absence of human emotions, and the higher cost.

The future of artificial intelligence

Artificial intelligence in health care may involve activities ranging from simple to sophisticated, including medical record review, population health trending and analytics, therapeutic medication and device design, radiological image interpretation, and clinical diagnosis and treatment.

## Conclusions

The path to artificial intelligence requires adequate training among dental professionals. The majority of institutions are currently unprepared for the dental and continuing education training that will be necessary for successful artificial intelligence integration in dentistry. Additionally, augmented reality (AR) and virtual reality (VR) both benefit from AI (AR). To improve learning and surgical planning, a unique idea known as mixed reality integrates components of generative artificial intelligence, VR, and AR into computer-generated information overlays. A future for artificial intelligence in the healthcare system cannot be ruled out given that multiple artificial intelligence systems for various dentistry specialties are being investigated and have demonstrated promising preliminary results. The potential for artificial intelligence systems to be a useful resource for oral health professionals is very high.
